# HIV-Associated Neurotoxicity: The Interplay of Host and Viral Proteins

**DOI:** 10.1155/2021/1267041

**Published:** 2021-08-25

**Authors:** Sushama Jadhav, Vijay Nema

**Affiliations:** ^1^Division of Molecular Biology, National AIDS Research Institute (ICMR-NARI), 73, G Block, MIDC, Bhosari, Post Box No. 1895, Pune, 411026 Maharashtra, India; ^2^Symbiosis International University (SIU), Lavale, Mulshi, Pune, 412115 Maharashtra, India

## Abstract

HIV-1 can incite activation of chemokine receptors, inflammatory mediators, and glutamate receptor-mediated excitotoxicity. The mechanisms associated with such immune activation can disrupt neuronal and glial functions. HIV-associated neurocognitive disorder (HAND) is being observed since the beginning of the AIDS epidemic due to a change in the functional integrity of cells from the central nervous system (CNS). Even with the presence of antiretroviral therapy, there is a decline in the functioning of the brain especially movement skills, noticeable swings in mood, and routine performance activities. Under the umbrella of HAND, various symptomatic and asymptomatic conditions are categorized and are on a rise despite the use of newer antiretroviral agents. Due to the use of long-lasting antiretroviral agents, this deadly disease is becoming a manageable chronic condition with the occurrence of asymptomatic neurocognitive impairment (ANI), symptomatic mild neurocognitive disorder, or HIV-associated dementia. In-depth research in the pathogenesis of HIV has focused on various mechanisms involved in neuronal dysfunction and associated toxicities ultimately showcasing the involvement of various pathways. Increasing evidence-based studies have emphasized a need to focus and explore the specific pathways in inflammation-associated neurodegenerative disorders. In the current review, we have highlighted the association of various HIV proteins and neuronal cells with their involvement in various pathways responsible for the development of neurotoxicity.

## 1. Introduction

Human immunodeficiency virus and acquired immunodeficiency syndrome (AIDS) are major public health problems globally, and HIV-1-associated dementia (HAD) is a momentous consequence of HIV infection [1]. Approximately 38 million individuals are presently infected with this virus and an average of one million individuals die each year from AIDS-related illnesses. At the same time, around 1.7 million individuals are getting newly infected while 26 million individuals were accessing antiretroviral therapy by the end of June 2020 [2]. The administration of combination antiretroviral therapy (cART) revolutionized the fight against AIDS and increased the number of people living with HIV along with the reduction in mortality rate transforming fatal disease conditions to 50% of people living with a chronic disease condition [3]. Even though HIV replication is affected by the use of cART, it does lead to the formation of latent reservoirs wherein the brain serves as an important reservoir for this virus. The persistence of the virus in the central nervous system directly results in distinct neurological symptoms such as neuroinflammation and neurotoxicity which indirectly increases susceptibility to cause opportunistic infections and HIV-associated neurocognitive disorders. This condition arises due to the infectious potential of various proteins of HIV as well [4]. Various cells from the central nervous system are getting affected following different pathways, and the level of toxicity varies as per the exposure of different viral proteins [5]. The immune response observed in terms of cytokine/chemokine production and antagonistic or neutralization effect developed against the action of various viral proteins to reduce neurotoxicity needs to be studied in detail. Thus, the review describes the various pathways and viral proteins involved in the development of HIV-associated neurocognitive disorder with special reference to HIV Nef virotoxin.

## 2. Structural Components and Replication of HIV

**Figure 1:**
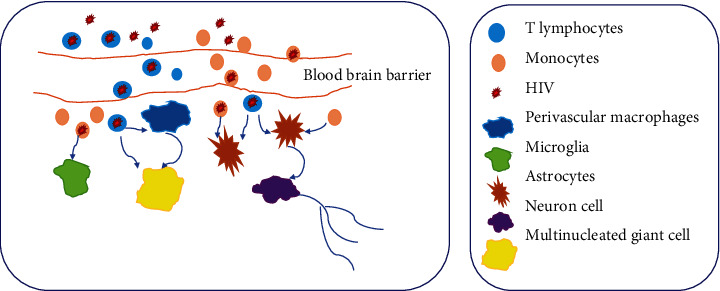
Representative images of HIV infection in various brain cells. HIV crosses the blood-brain barrier to go into the brain by means of infected macrophages, monocytes, and T lymphocytes and infects different types of brain cells.

The complete genome of the HIV-1 virus in the free-floating form is not infectious to the CD4 T cells; however, the replication of this virus in the CD4 cells followed by cell-to-cell transmission forming cellular chain reaction is an important event responsible for cell death [6]. Along with the CD4 receptor, the human immunodeficiency virus (HIV) requires a coreceptor for entry into target immune cells. The chemokine receptors CCR5 and CXCR4 are members of the G protein-coupled receptor superfamily. TheCCR5 and CXCR4 coreceptors have been identified as the principal coreceptors for macrophage-tropic and T cell line-tropic HIV-1 isolates, respectively. However, due to the attack of HIV on immune cells and their movement in the brain via the “Trojan horse” mechanism, it frequently compromises the integrity of the blood-brain barrier (BBB). Hence, the infection of the central nervous system (CNS) is observed in the early stages of HIV infection. Dysfunction of the BBB further potentiates viral replication within the CNS, which can lead to HIV-associated neuropathology. Apart from CD4 cells, the virus also infects monocytes/macrophages [7], dendritic cells [8], and microglia [9] with different mechanisms. As reported by Ances et al., microglial cells and macrophages support HIV infection in the brain dynamically, while infection of neurons and oligodendrocytes is seldom [4]. The HIV infection to astrocytes causing astrocytosis is also well reported [10]. The envelope glycoprotein gp120 and the transmembrane protein gp41 of an infectious HIV-1 particle are formed with the help of host cell lipid bilayer while spherical protein shell underneath known as matrix is in the inside layer. The subsequent inner layer of capsid protein (gag) is encapsulating two similar copies of the viral RNA inclusive of viral enzymes indispensable for HIV replication. These three enzymes are reverse transcriptase, integrase, and protease along with accessory as well as regulatory proteins (Tat, Rev, Nef, Vif, Vpu, and Vpr) that are essential during the formation of new viral copies [11]. Thus, HIV RNA and all the 15 proteins are essential structural components to form an infectious virion. These infectious viral copies are ready to infect CD4-positive immune cells and various other compartmental cells to infect the central nervous system (Figure 1).

## 3. Neurotoxicity Caused by Various HIV-1 Proteins

### 3.1. HIV Envelops Glycoprotein gp120

The glycoprotein gp120 is present on the outermost membrane of the HIV-1 viral particle. HIV gp120 plays a vital role in inducing neuronal injury and progressing the pathogenesis of HIV-associated neurocognitive disorder (HAND). Smith et al. reported that gp120 is a well-known potent neurotoxin causing neurodegeneration by means of direct and indirect pathways comprising interactions with coreceptors like CCR5 and CXCR4. Various pathways have been proposed indicating the role of HIV gp120 to trigger neurotoxicity via activation of macrophages and microglia as well as interaction with various chemokine receptors. Amongst these pathways, one is the binding of gp120 protein with various receptors expressed on the surface of the cells from CNS leading to production of reactive oxygen species and influx of intracellular calcium thereby activating cell signaling pathways indicating the development of neurotoxicity followed by cellular apoptosis. It is reported that the development of gp120-induced axonal toxicity could be completely prevented by blocking the CXCR4 receptor with monoclonal antibodies and partially for the CCR5 receptor [12]. Secondly, the interaction of gp120 to the N-methyl-d-aspartate (NMDA) receptor leads to excessive opening of N-methyl-d-aspartate receptor- (NMDAR-) gated cation channels, permitting the calcium ions' influx to toxic levels [13]. Thirdly, direct binding of HIV gp120 to either CCR5 or CXCR4 activates a p38-mitogen-activated protein kinase- (p38-MAPK-) mediated signaling cascade leading to neuronal apoptosis [14]. Fourthly, interaction between gp120 and CXCR4 also enhances the expression of the nicotinic receptor *α*7, which escalates influx of cellular [Ca2+] and leads to death of cells [15]. Additionally, one more important way is the direct interaction of HIV gp120 with neurons activating N-methyl-d-aspartate (NMDA) receptor by binding of glycine at glycine binding site thereby activating chemokine receptor. This interaction causes neuronal injury or neurotoxicity as evidenced by the mouse studies using NMDAR antagonists [16]. Hangxiu et al. explored in detail the role of HIV gp120 in promoting the trafficking and clustering of the NMDA receptors in membrane microdomains wherein it is mentioned that stimulation of gp120 leads to the development of signaling through the Src signaling pathway by a stabilized lipid raft complex. Thus, HIV gp120 interferes with NMDA receptor trafficking and plays a major role in synaptic dysfunction during the development of HIV infection [17]. Furthermore, the interaction of gp120 with receptors alters the membrane potential which is responsible for the regulation of excitatory synaptic transmission in neurons. Due to this, voltage- and ligand-gated ion channels get activated resulting in an inflow of calcium and sodium ions and efflux of potassium ions ultimately causing the death of cells [18].

### 3.2. HIV Transmembrane Protein gp41

Diagnosis of the neuropathological disorder is usually done based on the presence of multinucleated giant cells or gp41 or p24 antigen, and approximately one-fourth of the HIV-infected individuals develop dementia. Therefore, it is quite obvious that HIV-infected cells could be observed in higher amounts in the brains of some HAD patients than in the brains of people with HIV dying in the era of antiretroviral therapy. The transmembrane glycoprotein gp41 is present in the viral membrane, and during the process of fusion with the plasma membrane, the gp41 protein changes its conformation [19–21]. The expression of gp41 protein on an infected cell can interact with adjacent cells leading to an infection of a greater number of healthy cells which increases HIV viral load and is responsible for all the known adverse events including the development of dementia. Furthermore, Adamson et al. reported that there was an increased expression of gp41 and immunologic (type II) isoform of NO synthase (iNOS) simultaneously responsible for killing of neurons through a nitric oxide- (NO-) dependent mechanism and may lead to the major HIV-1-associated cognitive dysfunction [22]. Apart from this, gp41 is also responsible for the induction of matrix metalloproteinase activity in neuronal cultures leading to damaging of the neuronal connections [23]. Thus, there is an impact of gp41 glycoprotein in perturbance of neuronal activity following the development of neurotoxicity.

### 3.3. HIV Regulatory Protein Vpr

HIV-1 regulatory protein Vpr regulates the transcription process and enhances the production of virion [24]. Although HIV Vpr protein is reported to be present in the intracellular compartment, its presence is also reported in the cerebrospinal fluid of a patient suffering from HIV-associated neurocognitive disorder leading to the development of neurotoxicity [25]. The presence of Vpr protein in the extracellular fluids induces caspase-8 which further activates caspase-3, caspase-6, and caspase-7 contributing to death of cells. Moreover, the presence of Vpr protein leads to the formation of ionic conduits in the membrane of the neurons, with a large number of cations flowing in the inward direction leading to depolarization of the cell membrane and ultimately death of those cells [26, 27]. Thus, the presence of Vpr protein in the intracellular as well as extracellular compartment results in disturbance of neuronal signaling leading to induction of cell death.

### 3.4. HIV Regulatory Protein Tat

HIV-1 Tat protein is an imperative regulatory protein and plays an active role in the transactivation of the promoter region of the long terminal repeat which is vital for the expression of various genes and replication of HIV [28]. The human astrocytes are reported to take up the extracellular Tat protein secreted by HIV-infected cells in the *in vitro* study and localized it into the nucleus [29]. Interaction of Tat protein with various receptors like NMDA and non-NMDA such as glutamate receptors leads to excitotoxicity as well as activation of the sodium-potassium channel leading to increased permeability of cell wall and ultimately death of cells [30–32]. Furthermore, as reported by Buscemi et al., there is a direct and indirect neurotoxic effect of HIV Tat protein. The presence of HIV Tat protein leads to the death of neuronal cells due to immune activation and rapid as well as sustained production of cytokines mainly TNF*α* in macrophages and microglia [33]. It is observed experimentally that increased expression of TNF*α* and its receptor from patients with HAD has shown the synergistic effect of HIV Tat and TNF-*α* to promote neuronal death [34]. Along with the neurotoxic role of TNF-*α*, there are few more cytokines like IL-6, IL-8, IL-1b, and CXCL1 which secrete and exhibit Tat-mediated indirect neurotoxic effects. Apart from such neurotoxic factors, the heterogeneity in the *tat* gene also plays an important role, wherein due to the presence of critical CC motif in the tat protein, it recruits macrophages and monocytes into the brain and has a direct association with HAND. Neurotoxicity of Tat protein is also linked with the presence of R57 residue in the basic domain of *tat*, and polymorphisms at this juncture can show prominent effect on increasing the neurotoxic potential along with transactivation of the protein [35]. Enormous data is published in the literature with experimental pieces of evidence mentioning the involvement of HIV Tat and gp120 protein which cause neuronal injury and/or cellular apoptosis. An experimental study performed by Singh et al. in 2004 reported that after HIV infection, Tat and gp120 proteins induce striatal neurotoxicity through an apoptotic mechanism leading to gliosis and loss of neurons. These findings were confirmed by the in vitro analysis of the induced concentration of cytochrome C along with activation of caspase-3 and endonuclease G [36]. In the same year, Jana et al. reported that HIV gp120 and Tat induce the formation of ceramide, a chief inducer of apoptosis in human primary neurons. This ceramide is redox-sensitive and thereby activates neutral sphingomyelinases (NSMase). Additionally, gp120-coupled CXC chemokine receptor 4 (CXCR4) is involved in the induction of nicotinamide adenine dinucleotide phosphate (NADPH) oxidase-mediated production of superoxide radicals in neurons suggesting HIV gp120 mediated apoptosis of neurons in the CNS following the CXCR4-NADPH oxidase-superoxide-NSMase-ceramide pathway [37]. Further evidence of HIV Tat-mediated neuronal apoptosis is presented by Agrawal et al. in 2007 wherein they checked the activity of Cu/Zn superoxide dismutase (SOD1) and glutathione peroxidase (GPx1), which are antioxidant enzymes, to understand signaling pathways and neuroprotective activity for apoptosis induced by Tat. This study highlighted the involvement of Tat in the activation of multiple signaling pathways leading to neuronal apoptosis [38]. Subsequently, a review published by Scutari et al. in 2017 reported the involvement of various HIV proteins in the stimulation of cell signaling pathways in the control of cell survival and apoptosis. It further highlighted various parameters linked with the inception of neurocognitive disorders and talk about formulating the novel therapeutic approaches to prevent reproduction of HIV in the CNS [39]. Considering the published literature, it is evident that various mechanisms are followed leading to cellular apoptosis significantly with differences in the mechanism of action between HIV gp120 and Tat proteins [40].

### 3.5. HIV Regulatory Protein Rev

HIV Rev protein is also one of the important regulatory proteins during the transcription step of the virus. This protein is playing an important role in controlling the splicing activity and transportation of the viral RNA from the nucleus of the cells towards the outer cytoplasmic compartment. This protein is also important in the translation of the transcripts [41]. An *in vivo* study carried out in mice using Rev protein could not differentiate the direct and indirect neurotoxic effects on cells, and more research needs to be done for understanding the role of this protein in the development of neurotoxicity [42].

### 3.6. HIV Membrane Protein Vpu

The protein Vpu is mainly present in the intracellular compartment and is an integral part of the membrane protein. This protein is majorly involved in strengthening the release of viral copies from the HIV-1-infected cells [43]. Although this protein is not detected in the various compartments of the brains of HAD patients, its toxicity cannot be ruled out due to its involvement in the formation of the ion channels and increasing the permeability of lipid bilayer for sodium and potassium ions facilitating cell death [44].

### 3.7. HIV Regulatory Protein Nef

HIV Nef protein is a regulatory protein responsible for the enhancement of viral replication, antigen presentation, and immune evasion [45]. Maintenance of high viral load and appropriate progression to AIDS can be possible due to the presence of HIV Nef protein by interfering with the regular trafficking pathway of MHC-I and further reducing recognition of CTL response and lysis of infected cells [46]. The currently available literature indicates that the presence of HIV Nef protein is either as a membrane fusion protein or released in an extracellular compartment through exosomes while it affects the intracellular trafficking of various distinct pathways through sharing of few common elements [47] while *in vitro* experiments have shown that Nef protein may be expressed on the outer surface of infected peripheral blood mononuclear cells (PBMCs) and T lymphocytes [48]. Furthermore, Nef is responsible for the activation of caspases, free radicals, and membrane potential leading to depolarization of the membrane. All the evidence indicates the role of Nef at a different level, and a better understanding is essential to develop a drug that will increase anti-HIV activity and control the rate of disease progression [46].

## 4. Type of Cells Infected with HIV in the Central Nervous System (CNS)

### 4.1. Microglial Cells

Microglial cells play an active role in developing immune responses against infectious agents as well as the progression towards neuroinflammation stage and neurological disorders like Parkinson's, Alzheimer's, and Huntington's disease and even HAND. It is reported that though HIV does not harm neurons directly by productive infection, it harms by promoting the production of inflammatory factors and reactive oxygen species (ROS) from infected microglia and macrophages [40]. Microglia possess CCR3 and CCR5 coreceptors on their surfaces and hence are susceptible to HIV infection, further leading to the development of HIV-associated dementia [49, 50]. Furthermore, infection in microglia can remain in latent form for several months or years due to the expression of host restriction factor, namely, tetherin [51, 52]. Neuronal damage and ultimately neuronal apoptosis are reported due to overactivation of microglia in HIV-associated neurocognitive disorder leading to induction of proinflammatory chemokines and cytokines [53]. Activation of the autophagy pathway is an important phenomenon that occurs when HIV replication starts in microglia thereby activating the cells to produce various cytokines like IL6, IL8, TNF-*α*, CCL2, and RANTES [54]. Hallmarks of the neuropathology associated with HIV infection and HAND/HAD are the formation of multinucleated giant cells as well as microglial nodules. As reported by Botticelli et al. in 2004, the evidence of multinucleated giant cells and microglial nodules has been detected after histopathological analysis from brain tissue of the AIDS patients, as the microglial cells were positive for immune-histochemical markers like Ricinus communis agglutinin-1 (RCA 1) and ferritin while the absence of these markers in other types of cells from the brain confirms the source of multinucleated giant cells from microglia [55]. RCA-1 stain for microglia while stained ferritin shows the presence of free ferritin and is an indicator of neurodegeneration. Subsequently, Nebuloni et al. also reported the presence of multinucleated giant cells and p24 antigen in the brains of HIV/AIDS patients [56]. A report published by Streit in 2004 mentioned that activated microglia are the neurotoxin-producing immune effector cells which are actively involved in developing neurodegeneration leading to cause Alzheimer's disease dementia while Tai et al. in 2007 correlated the role of activated microglia in striatal neuronal dysfunction with the preclinical Huntington's disease [57, 58]. Furthermore, the activated microglia are reported to be a chronic source of multiple neurotoxic factors responsible for damaging neurons progressively. Thus, HIV does not harm neurons directly by productive infection, but rather, it harms by promoting the production of inflammatory factors like tumor necrosis factor-*α*, nitric oxide, interleukin-1*β*, and reactive oxygen species (ROS) from infected macrophages and microglia [40, 59].

### 4.2. Astrocytes

Astrocytes play vital roles in the CNS including maintenance of homeostasis, providing structural support to neurons, and maintaining proper functioning of the nervous system by inactivation of neurotransmitters and preserving extracellular potassium levels [60]. Churchill et al. reported that astrocytes are latently infected and do not produce new viral particles. They also reported that in individuals with HIV-associated dementia, astrocytes play a major role in placement of monocytes secreting many chemokines which includes mainly CCL2, CCL3, CCL5, CXCL10, and CX3CL1, 2. This indicates that astrocytes infected with HIV play a dynamic role in placement of monocytes across the BBB. The experimental evidence indicates the magnitude of astrocyte infection leading to neuropathogenesis, and future studies are essential to understand the pathological mechanisms of neurodegenerative diseases [61]. Boutet et al. highlighted the expression of CCR5, CCR3, and CXCR4 transcripts on embryonic astrocytes and microglial cells while there is absence of CD4 receptor at cell surface level of purified astrocytes. Hence, despite the presence of the CCR5 receptor, the R5 strain of HIV cannot enter into astrocytes. Moreover, experimental evidence of M tropic isolates from the brain cells suggests the utilization of an alternative coreceptor for viral entry [62].

Alternative mechanisms of astrocyte infection are reported in published literature wherein astrocytes can also get infected with HIV either due to endocytosis mechanism or engulfing of HIV-infected macrophages or activation with a stimulant like proinflammatory cytokines [63, 64]. Though replication of HIV is restricted in astrocytes, the cells release HIV proteins and induce inflammation to cause neuronal damage [65, 66] while sometimes HIV can remain in a latent stage in astrocytes at the epigenetic level [67]. Amongst the various viral proteins, HIV Tat activates transcription and enhances infection of HIV in astrocytes while HIV Nef can encourage ROS production and promote rapid neuronal death leading to the development of HAD [68]. Furthermore, it is reported that HIV-1-infected astrocytes can modify the blood-brain barrier (BBB) due to epithelial cell apoptosis [69].

### 4.3. Oligodendrocytes

There is an absence of CD4 and CCR5 expression but presence of CXCR4 coreceptor expression on the surface of oligodendrocytes [70]. These cells get damaged due to the secretion of viral proteins from the virion itself or other infected cells of the CNS [71, 72]. HIV-1 viral protein Tat can cause significant neurotoxicity by modifying the balance of CaMKII_ and glycogen synthase kinase 3_ (GSK3_) leading to apoptosis of oligodendrocytes and development of HIV-associated neuropathology while HIV Tat protein can also interact with NMDA receptor that leads to induction of Ca2+ and Ca2+/calmodulin-dependent protein kinase II (CaMKII_) leading to the death of oligodendrocytes [73, 74]. It is necessary to explore the impact of all different viral proteins on different types of cells from the central nervous system in the future.

### 4.4. Perivascular Macrophages

These are important cells in forming a main part of the blood-brain barrier (BBB) and are mainly located in perivascular spaces of the brain. These cells express CD4 receptor and CCR5 and CXCR4 coreceptors on their surface. Their major function is in communicating messages between the immune system and the brain as well as in the transmission and modulation of inflammatory signals from the periphery [75]. However, reactive oxygen species (ROS) and nitric oxide are produced by macrophages and that interferes with associated signaling pathways leading to apoptosis and causing cell cycle arrest as well as severe damage to DNA and proteins [76]. Macrophages are the principal target cell and mediator of neuronal injury in modifying the production of astrocyte CXCL8 in HIV-associated dementia. The production of CXCL8 was dependent on the production of monocyte-derived macrophages (MDM), IL-1*β*, and TNF-*α* following viral and immune activation. Additionally, high levels of IL-8 increase replication of HIV-1 in CD4 T cells and macrophages in HIV-associated dementia resulting in the release of ATP and glutamate leading to neurotoxicity [77–79].

### 4.5. Neurons

These are the elementary functional components of the nervous system responsible for electrical excitation and transmission of information [80]. Few studies have reported that the mature neurons express CD4, CCR5, CXCR4, and CCR3 coreceptors on their surfaces, while expression of the CD4 receptor is controversial. Moreover, neurons seem to be overall resistant to HIV infection, but can take up viral proteins, which leads to neuronal apoptosis. At the same time, Bissel et al. reported that CD4-independent mechanisms have been used for the entry of HIV in the neurons [81–83]. Replication of HIV and the release of viral proteins damage the functioning of neurons leading to their death. In particular, studies have reported about HIV Nef and Tat proteins wherein HIV viral protein Nef prevents autophagic maturation leading to neurodegeneration [84] while Tat protein activates forkhead box O3 (FOXO3) leading to induction of neuronal apoptosis [85]. Furthermore, it is reported by Kaul et al. that HIV-1, as well as its structural proteins mainly envelope protein, interferes directly affecting biological functions of neural stem and progenitor cells via the receptor-ligand axis between CXCR4-SDF-1 causing neuronal injury followed by the death of existing neurons leading to the development of HAD. Thus, there is disturbance of the potential homeostasis induced due to virus and mechanisms of renewal in the CNS [86].

## 5. Entry of HIV in the Central Nervous System (CNS) and Disease Development

Till now, we have seen that HIV enters in various types of cells and viral protein plays important role in the development of neurotoxicity. However, based on the use of combination antiretroviral therapy (cART) along with signs and symptoms of the individual, the severity of the disease is categorized. The drop-in functional activity of the brain and behavioral changes after exposure to HIV infection contribute in the development of HIV-associated neurocognitive disorder, and considering the severity of symptoms, this disorder is categorized into asymptomatic neurocognitive impairment (ANI), mild neurocognitive disorder (MND), and HIV-associated dementia (HAD) [87]. HAD is the severe form of all these different categories, and it was defined well before the start of cART therapy in 2004 as symptoms related to deterioration in the functioning of the brain were already well characterized. Due to the implementation of cART therapy to individuals living with HIV, there seems to be an improvement in the functioning of the brain and it reduced this condition by up to 40-50% [88]. The infection of other viruses along with HIV infection and the presence of comorbidities of an individual can lead to an increase in mental depression up to 70% and thereby increasing neurological complications [89].

## 6. Association of Various Pathways with Neurotoxicity

**Figure 2:**
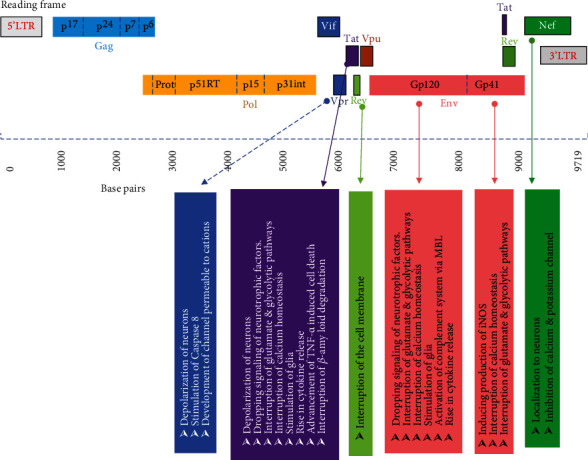
The impact of HIV viral proteins affecting various mechanisms.

It has been reported that many neurological symptoms are expressed by individuals suffering from human immunodeficiency virus type 1 (HIV-1) infection. HIV-1 is mostly affecting motor and cognitive functions and ultimately reaches a stage of devastating HIV-associated dementia (HAD). Being a complex phenomenon, it is very difficult to understand HIV-related neurotoxicity, and various mechanisms are getting affected directly by various viral proteins and indirectly by the impact on various host proteins such as cytokines and chemokines. During replication of the virus, the structural genes like gp120 and gp41 and accessory genes like Tat, Nef, and Rev get activated and these proteins are released in the extracellular space. The impact of the damage on the neuronal cells is dependent on the replication-competent status of the viral particle or secreted viral proteins. Usually, HIV-1 Tat and gp120 proteins are neurotoxic following either an oxidative stress pathway or dopaminergic pathway [90]. Additionally, damage due to Tat protein is associated with an increase in apoptosis, altered calcium homeostasis, stimulation of NF-Kb and TNF-*α*, activation of nitric oxide synthase, stimulation of glutamate receptors, and production of nitric oxide [91–94]. A similar kind of neurotoxicity had been observed due to gp120 protein affecting these pathways as well [95]. To prevent the impact of such toxicity, certain therapeutic agents have been developed which would help in reducing the toxicity of these viral proteins and improving the status of HIV-associated dementia [96].  Figure 2 describes the affected mechanisms due to various viral proteins such as interruption of ion potential, depolarization of neurons, stimulation of complement system via increasing the expression of mannose-binding lectin, and induction of apoptosis.

Various in vitro and in vivo studies correlate neurodegeneration developed due to HIV-induced inflammation with the production of proinflammatory cytokine/chemokine, excitotoxic neuronal injury, and oxidative stress [97, 98]. Amongst these, the production of alpha and beta chemokines is playing a major role in the development of neurotoxicity. As reported by Geissmann et al. in 2003, chemokine CX3CL1 from the CX3C chemokine family attaches to endothelial cells facilitating attachment of monocyte thereby enhancing migration of monocyte across the blood-brain barrier as well as into the CNS. This phenomenon is commonly associated with an increase in inflammation in the brain and is observed in patients with HIV infection [99–101]. Furthermore, it is reported that upregulated expression of CXCL1 fractalkine is observed in patients suffering from AIDS dementia complex (ADC) as well as in in vitro cultures of HIV-infected astrocytes and macrophages [102, 103].

### 6.1. Association of Kynurenine and NMDAR Pathways with Neurotoxicity

Amongst 9 essential amino acids, tryptophan is one of the essential amino acids for various functions. This tryptophan is catabolized with the help of various enzymes under the kynurenine pathway. The end product of this pathway is nicotinamide adenine dinucleotide (NAD) which is a cofactor central to metabolism. The major metabolites of the kynurenine pathway are tryptophan, kynurenine, kynurenic acid, xanthurenic acid, quinolinic acid, and 3-hydroxykynurenine [104]. Major functions of tryptophan are like production of energy, metabolism, immune tolerance, neurotoxicity, and protein, serotonin, and melatonin synthesis. In the kynurenine pathway, the first and rate-limiting enzyme is indoleamine 2,3-dioxygenase (IDO). It is reported that there is a reduction in T cell response to various antigens if IDO is overexpressed [105]. The activity of IDO is responsible for the creation of a tryptophan-depleted microenvironment resulting in modulating protein synthesis and also downregulating the proliferation of T cells [106, 107]. The IDO enzyme essentially acts as a catalyst during the conversion of tryptophan to N-formylkynurenine followed by the production of kynurenine as an intermediate metabolite and then the production of QUIN. QUIN is one of the neurotoxins acting through the NMDA receptor and lipid peroxidation. The different cell types in the brain except monocytic lineage cells do not facilitate the full complement of the kynurenine pathway and hence are not responsible for the production of QUIN [104]. Although microglia and macrophages do synthesize QUIN, astrocytes do not synthesize QUIN due to the lack of kynurenine hydroxylase enzyme within the CNS [108]. However, astrocytes can produce kynurenine which is converted to QUIN by the surrounding macrophages and microglia. QUIN increases the production of chemokine (MCP-1) thereby allowing entry of activated monocytes, T lymphocytes, and intrinsic neuronal cells with major expression of CCR5 receptors and infecting various types of cells from the brain with HIV virus [109]. HIV-infected macrophages and microglia activate the kynurenine pathway leading to immune tolerance and HIV-associated neuropathogenesis. Thus, activation of the kynurenine pathway and production of QUIN lead to excitotoxicity through activation of NMDA receptors on astrocytes as well as on neurons. Further studies are essential to understand the role of kynurenine components and the association of viral proteins in HIV-associated neurocognitive disorders.

## 7. Conclusions and Future Perspectives

This review highlighted the role of viral proteins and cellular factors involved in the development of neurotoxicity leading to the cause of HIV-associated neurocognitive disorder. It also focuses on the mechanisms getting affected due to infection of HIV and activation of various pathways along with the development of neurotoxic products encoded by the host genome. Further, the role of specifically kynurenine and NMDAR pathways and their association with neurotoxicity responsible for neurodegeneration are discussed. This information is necessary to understand the importance of essential amino acid metabolism and their mechanism of action providing evidence for the development of HIV-associated neurocognitive disorder. Newer antiretroviral therapies are on the rise and reduce the burden of HIV infection. However, with longer survival of the infected individuals, there is a development of a neurocognitive disorder which needs to be focused on in the future. Newer interventions targeting these disorders may further support the quality of life in HIV-infected individuals.

## Abbreviations

CNS: Central nervous system
BBB: Blood-brain barrier
NMDAR: N-Methyl-d-aspartate receptor
ADC: AIDS dementia complex
QUIN: Quinolinic acid
MDM: Monocyte-derived macrophages
ROS: Reactive oxygen species
NAD: Nicotinamide adenine dinucleotide
ANI: Asymptomatic neurocognitive impairment
MND: Mild neurocognitive disorder
HAD: HIV-associated dementia
IDO: Indoleamine 2,3-dioxygenase
MAPK: Mitogen-activated protein kinase
RCA 1: Ricinus communis agglutinin-1.
